# Cancer-associated fibroblasts from cervical cancer modulate protumoral neutrophil functions: evidence from a pilot exploratory study

**DOI:** 10.3389/fimmu.2026.1900567

**Published:** 2026-10-09

**Authors:** Luis Javier Reséndiz-Castillo, Marcela Guadalupe Martínez-Barajas, Ahtziri Socorro Carranza-Aranda, Jonathan René García-Bernal, Carlos Daniel Díaz-Palomera, Adrián Ramírez-de-Arellano, Moisés Ramos-Solano, Natali Vega-Magaña, Julio César Villegas-Pineda, Ana Laura Pereira-Suárez

**Affiliations:** 1 Universidad de Guadalajara, Centro Universitario de Ciencias de la Salud, Departamento de Microbiología y Patología, Instituto de Investigación en Cáncer e Infecciones, Guadalajara, Jalisco, Mexico; 2 Universidad de Guadalajara, Centro Universitario de Ciencias de la Salud, Departamento de Disciplinas Filosóficas, Metodológicas e Instrumentales, Guadalajara, Jalisco, Mexico; 3 Universidad de Guadalajara, Centro Universitario de Ciencias de la Salud, Departamento de Biología Molecular, Guadalajara, Jalisco, Mexico

**Keywords:** cancer-associated fibroblasts, cervical cancer, tumor immunology, tumor microenvironment, tumor-associated neutrophils

## Abstract

Cancer-associated fibroblasts (CAFs) are key regulators of the tumor microenvironment; however, their role in modulating neutrophil function during cervical cancer (CC) progression remains poorly understood. In this study, we evaluated whether CAF supernatants (CSN) derived from early (FIGO I–II) and late stages (FIGO III–IV) of CC differentially regulate neutrophil activity. Peripheral blood neutrophils from healthy donors were stimulated with CSN, and their functional responses were assessed, including cytokine production, metabolic activity (MTT), reactive oxygen species (ROS) generation, apoptosis and survival analyses, expression of activation (CD66b) and protease degranulation markers (MMP9 and NE), and neutrophil extracellular trap (NET) release. CSN factors promoted a predominantly proinflammatory cytokine profile in neutrophils, characterized by increased production of IL-6, IL-8, TNF-α, and IFN-γ. Additionally, stimulation with CAF enhanced neutrophil metabolic activity, ROS production, survival, and expression of CD66b, MMP9, and neutrophil elastase, suggesting preliminary stage-associated differences in neutrophils responses between CAFs derived from early- and late-stage tumors. Notably, NET formation was significantly higher in neutrophils stimulated with early-stage CAFs, whereas late-stage CAFs induced a metabolically active but lower NETosis release. These exploratory findings suggest that cervical cancer-derived CAFs may differentially modulate neutrophil functional responses according to tumor stage, highlighting the CAF-neutrophil axis as a potentially relevant component of the cervical cancer microenvironment.

## 1 Introduction

Cervical cancer (CC) ranks as the fourth most common malignancy in women worldwide, with an estimated 662,301 new cases and 348,709 deaths in 2022 (1, 2). Treatment and prognosis are based on the Federation of Gynecology and Obstetrics (FIGO) staging system; however, clinical progression varies considerably even among patients with the same stage, suggesting that tumor evolution is also shaped by stromal and immune cell populations within the tumor microenvironment (TME) (3). The neutrophil-to-lymphocyte ratio (NLR) has emerged as a robust prognostic biomarker, with elevated values consistently associated with worse survival outcomes across solid tumors (4, 5). Especially in cervical cancer, multiple meta-analyses have consistently shown that an elevated NLR before treatment is an independent predictor of poorer survival and is associated with late FIGO stage and lymph node involvement (6–8).

Cancer-associated fibroblasts (CAFs) constitute the most abundant stromal population in solid tumors and are recognized as central modulators of immune cell function through the secretion of soluble factors from CAFs, such as growth factors, cytokines, and chemokines (9, 10). Recent studies have demonstrated that CAFs from hepatocellular carcinoma and pancreatic cancer reprogram neutrophils by inducing chemotaxis, protecting them from apoptosis, and increasing expression of activation markers such as CD66b and PD-L1 through the IL-6/STAT3 pathway (11). Moreover, CAF-derived signals can promote neutrophil activation and polarization into a protumoral functional activity through the secretion of cytokines such as IL-8 (12) and growth factors like TGF-β (13, 14).

In this context, neutrophils, the most abundant circulating immune cells, play roles that extend well beyond antimicrobial defense, including regulation of inflammation and active participation in cancer progression (15, 16). Within the TME, recruited neutrophils are called tumor-associated neutrophils (TANs). Importantly, these TANs exhibit functional plasticity, polarizing toward antitumoral (N1) or protumoral (N2) phenotypes in response to microenvironmental cues such as TGF-β and IFN-γ (17, 18). These N2 protumoral neutrophils deploy effector mechanisms including enhanced neutrophil survival, reactive oxygen species (ROS) production (19), proinflammatory cytokine secretion (20), stimulated release of granule proteases including neutrophil elastase (NE) and Matrix Metalloproteinase-9 (MMP9) (21), and induced NETosis release (17, 18, 22, 23). In this context, NETs, originally described as antimicrobial structures, have been implicated in promoting metastasis, cancer-associated thrombosis, and immune evasion in the tumoral context (13, 14).

Despite these advances, the interaction between CAFs and neutrophils in cervical cancer remains unexplored. Other studies on the cervical immune microenvironment have focused on macrophages (24), T cells (25, 26), and myeloid-derived suppressor cells (27). In this study, we explored whether CSN derived from patients with different FIGO stages of CC modulates neutrophil activity and functional polarization, including cytokine secretion, metabolic activity, ROS production, expression of activation markers, and NET formation.

## 2 Materials and methods

### 2.1 Patient characteristics

Patients diagnosed with CC were recruited at the High Specialty Medical Unit, Gynecology and Obstetrics Hospital of the Mexican Institute of Social Security (IMSS) between June 2024 and June 2025. Tumor staging was determined according to the FIGO classification system by the pathology service. Clinical data, including age, histological subtype, clinical stage, and relevant medical history, were collected from medical records. Only patients who had not received prior chemotherapy or radiotherapy were included in the study. Neutrophil-to-lymphocyte ratio (NLR) was calculated as the ratio of absolute neutrophil and lymphocyte counts obtained from peripheral blood hematological biometry.

### 2.2 Fibroblast isolation

Fibroblasts were isolated from six tumor biopsies, including three from early-stage FIGO (I–II) and three from late-stage FIGO (III–IV). Tumor samples obtained from patients with cervical carcinoma were mechanically dissociated using a scalpel in a sterile Petri dish. Subsequently, small tissue fragments (1–2 mm) were enzymatically digested using a mixture of collagenase I and dispase (0.1%, 0.75 μg/μL) for 24 h at 4 °C. Samples were then incubated in 1 mL of trypsin-EDTA (Cat. No. T4049, Sigma-Aldrich, St. Louis, MO, USA) for 5 min. After discarding the supernatant, digested cells were cultured in DMEM/F12 (Cat. No.10565018, Gibco™, Waltham, MA, USA) medium supplemented with 10% heat-inactivated fetal bovine serum (FBS; Cat. No. 16000044, Gibco™, Waltham, MA, USA) and 1% antibiotic/antimycotic (Cat. No.15140122, Gibco™, Waltham, MA, USA). Cells were maintained in a humidified atmosphere at 37 °C with 5% CO_2_ for 2 h. After this period, the medium was replaced with fresh supplemented medium. Cell morphology was periodically monitored by phase-contrast microscopy.

### 2.3 Immunophenotypic characterization of CAFs

Fibroblasts were characterized by immunofluorescence using CAF markers FAP, α-SMA, and vimentin. Briefly, 5,000 cells per well were seeded in chamber slides. After 24 h, cells were washed with PBS and fixed with 4% paraformaldehyde for 15 min at room temperature (RT).

Cells were permeabilized with 0.2% Tween-20 (Cat. No. 1706531, Bio-Rad, Hercules, CA, USA) for 10 min and blocked with PBS containing 10% FBS for 1 h at RT. Primary antibodies against vimentin (Mouse IgG; Cat. No. ab8978, Abcam, Cambridge, MA, USA.; 1:500), α-SMA (Mouse IgG; Cat. No. ab7817, Abcam, Cambridge, MA, USA; 1:500) and FAP (Rabbit IgG; Cat. No. ab28244, Abcam, Cambridge, MA, USA; 1:200) were incubated overnight at 4 °C.

Secondary antibodies Alexa Fluor 488 goat anti-mouse IgG1 (Cat. No. A-21121, Invitrogen, Waltham, MA, USA; 1:1000) and Alexa Fluor® 647 goat anti-rabbit IgG (Cat. No. ab150083, Abcam, Cambridge, UK; 1:1000) were incubated for 2 h at RT. Nuclei were counterstained with DAPI using VECTASHIELD mounting medium (Cat. No. H-1200-10, Vector Laboratories Inc., Newark, CA, USA). Fluorescence images were acquired using an Axio Imager A2 microscope (Carl Zeiss AG, Jena, Germany) and analyzed with ZEN software (version 2.1.57.1000 Lite Blue edition).

### 2.4 CAFs supernatant collection

Once fibroblasts were characterized as CAFs, they were cultured in T-75 culture flasks until 90% confluence was reached. At this point, the supplemented medium was replaced with serum-free medium for 24 h. CSNs were then collected, filtered through a 0.22 μm filter, and stored at −70 °C until use. Additionally, a non-tumorigenic human lung fibroblast cell line (CCD-25Lu, ATCC, Manassas, VA, USA) was used as a control representing non-tumorigenic fibroblast reference to provide a non-cancerous fibroblasts comparison, CCD-25Lu was cultured in EMEM (Cat. No. 30-2003, ATCC, Manassas, VA, USA) medium supplemented with 10% heat-inactivated fetal bovine serum (FBS; Cat. No. 16000044, Gibco™, Waltham, MA, USA) and 1% antibiotic/antimycotic (Cat. No.15140122, Gibco™, Waltham, MA, USA). Cells were incubated at 37 °C, 5% CO2 until 90% confluence was reached.

### 2.5 RNA extraction and transcriptomic analysis

For transcriptomic analysis, total RNA was extracted from CAFs using the RNeasy Plus Mini Kit (Cat. No. 74634, Qiagen, Venlo, The Netherlands) according to the manufacturer’s instructions. RNA concentration and purity were assessed by absorbance at 260/280 nm and 260/230 nm to evaluate protein and phenolic contamination, respectively. Samples were preserved at −80 °C in GenTegra RNAssure elution tubes (Cat. No. GTR50-LQ, GenTegra, Pleasanton, CA, USA) until further processing. RNA next-generation sequencing was performed on the NovaSeq 6000 platform by Novogene Bioinformatics Technology Co., Ltd. (Sacramento, CA, USA). Raw sequencing data were analyzed on the open-source Galaxy platform (v0.12.1), and sequence quality was assessed using FastQC (v0.12.1). Differentially expressed genes (DEGs) were identified in RStudio (R v4.5.2) using the DESeq2 package (v1.50.2). Raw count matrices were normalized using the median-of-ratios method, and log2 fold change (log2FC) estimates were improved using the apeglm shrinkage estimator. Genes were considered differentially expressed using a cutoff of |log2FC| ≥ 1 and an adjusted p value (padj) < 0.05 according to the Benjamini–Hochberg correction method. Functional enrichment analyses were performed using the list of differentially expressed genes (DEGs). Gene Ontology (GO) and Kyoto Encyclopedia of Genes and Genomes (KEGG) pathway enrichment analyses were conducted in RStudio using the clusterProfiler package (v4.16.0). GO terms and KEGG pathways with an adjusted p value < 0.05 were considered significantly enriched. Gene Set Enrichment Analysis (GSEA) was performed using the GSEA Desktop application (Broad Institute, Cambridge, MA, USA) in Preranked mode with the Hallmark gene set collection (h.all.v2023.2.Hs.symbols.gmt) obtained from the Molecular Signatures Database (MSigDB). Genes were ranked according to the DESeq2 Wald statistic, and 1,000 phenotype permutations were performed. Gene sets with a false discovery rate (FDR) < 0.25 were considered significantly enriched, following the recommendations of the Broad Institute. Enrichment results were visualized as bubble plots using ggplot2 in R.

### 2.6 Neutrophil isolation and stimulation

Peripheral blood from clinically healthy women donors was collected in BD Vacutainer® EDTA-containing tubes (Cat. No. 367863, Becton Dickinson, East Rutherford, NJ, USA). Neutrophils were isolated using a double-density gradient, with Histopaque®-1119 (Cat. No.11191, Sigma Aldrich, St. Louis, MI, USA) for the bottom layer and Histopaque®-1077 (Cat. No.10771, Sigma Aldrich, St. Louis, MI, USA) for the top layer, followed by centrifugation at 890 × g for 30 min.

Plasma and PBMC fractions were discarded, and granulocytes were collected from the lower layer. Cells were washed with sterile saline buffer and resuspended in RPMI-1640 (Cat. No. 11875093, Gibco™, Waltham, MA, USA) supplemented with 10% FBS and 1% antibiotic/antimycotic. Viability was assessed using trypan blue exclusion. Neutrophils were isolated from independent healthy donors for each biological replicate. For experiments, 200,000 neutrophils per well were seeded and allowed to stabilize for 30 min at 37 °C and 5% CO_2_. Neutrophils were stimulated with CSNs from different clinical stages, early and late, and CCD-25Lu supernatants at a 1:2 ratio. Lipopolysaccharide (LPS, 100 ng/mL; Cat. No. L3129, Sigma Aldrich, St. Louis, MI, USA) was used as a positive control, and untreated neutrophils as a negative control.

### 2.7 Cytokine profile of stimulated neutrophils

Cytokine production was determined using a multiplex immunoassay (Cat. No. M5000031YV, Bio-Plex Pro™ Human Cytokine 8-plex Assay, Bio-Rad) that measured GM-CSF, IFN-γ, IL-2, IL-4, IL-6, IL-8, IL-10, and TNF-α. Cytokines were measured following the manufacturer’s instructions. Each cytokine concentration was determined using the MAGPIX® flow system (Bio-Rad, Hercules, CA, USA) and was expressed in pg/mL. Assays were performed according to the manufacturer’s instructions, and all samples were analyzed in triplicate in a single experiment.

### 2.8 MTT assay for neutrophil metabolism

Neutrophil metabolic activity was assessed using the MTT assay. A total of 2 × 10^5^ cells per well were seeded in 96-well plates and stimulated with CSN or CCD-25Lu fibroblast supernatants for 24 h. LPS was used as a positive control, while untreated neutrophils served as a negative control. MTT solution (Cat. No. M5655, Sigma Aldrich, St. Louis, MI, USA) at a concentration of 5 mg/mL was added to cells (10 μL per well) and incubated for 3 h at 37 °C with 5% CO_2_. Formazan crystals were dissolved in 100 μL DMSO (Cat. No. D8418, Sigma Aldrich, St. Louis, MI, USA), and absorbance was measured at 570 and 690 nm using a Multiskan™GO plate reader (Thermo Scientific™, Waltham, MA, USA). Experiments were performed in triplicate across three independent experiments.

### 2.9 Measurement of reactive oxygen species production

ROS production was measured in stimulated neutrophils (200,000 cells/well) for 24 h, then incubated with 20 μM DCFDA for 30 min at 37 °C using the DCFDA/H2DCFDA solution (Cat. No. ab113851, Abcam, Cambridge, MA, USA). TBHP (55 μM) was used as an internal control, LPS as a positive control, and untreated neutrophils as a negative control. Cells were washed and resuspended in buffer with 10% FBS. Fluorescence was measured at 485 nm excitation and 535 nm emission using a Synergy HTX microplate reader (BioTek, Winooski, VT, USA). Results were expressed as relative fluorescence units (RFU). Experiments were performed in triplicate across three independent experiments.

### 2.10 Flow cytometry of cell apoptosis

Neutrophils (300,000 cells/well) were stimulated for 24 h, after that period, cells were collected and stained to assess apoptosis using the FITC Annexin V and propidium iodide (PI) kit (Cat. No. V13242, Invitrogen™, Waltham, MA, USA). Positive controls for death were included in neutrophils treated with 2% DMSO (apoptosis) and 5% DMSO (necrosis). Cells were stained with Annexin V and propidium iodide (PI) for 15 min at RT, then resuspended in PBS and analyzed using an Attune™ NxT Acoustic Focusing Cytometer (Invitrogen™, Waltham, MA, USA). A total of 10,000 events per sample were acquired using BL1-A (530 nm) and BL2-A (>575 nm) detection filters. Data were analyzed using FlowJo™ software (version 10.5.3, FlowJo LLC, Ashland, OR, USA). Experiments were performed in triplicate across three independent experiments. Doublets were excluded based on forward scatter height versus area, and debris was excluded using forward and side scatter parameters. Compensation controls were included for fluorescence overlap correction.

### 2.11 Immunofluorescence of neutrophil activation markers

Neutrophils (200,000 cells) were cytospun onto glass slides and stimulated for 24 h. Cells were fixed with 4% paraformaldehyde, permeabilized with 0.2% Tween-20, and blocked with 10% fetal bovine serum (FBS). Primary antibodies anti-CD66b (Mouse IgG; Cat. No. 392902, BioLegend®, San Diego, CA, USA; 1:200), anti-Neutrophil Elastase (NE) (Rabbit IgG; Cat. No. MA5-32548, Invitrogen™, Waltham, MA, USA; 1:200), and anti-Matrix Metalloproteinase-9 (MMP9) (Mouse IgG; Cat. No. ab76003, Abcam, Cambridge, UK; 1:500) were incubated overnight at 4 °C.

Secondary antibodies Alexa Fluor™ 488 goat anti-mouse IgG1 (Cat. No. A-21121, Invitrogen™, Waltham, MA, USA; 1:1000) and Alexa Fluor™ 488 goat anti-Rabbit IgG (Cat. No. A-32731, Invitrogen™, Waltham, MA, USA; 1:1000) were incubated for 2 h at RT. Nuclei were counterstained with DAPI using VECTASHIELD® mounting medium (Cat. No. H-1200-10, Vector Laboratories Inc., Newark, CA, USA). Fluorescence images were acquired using an Axio Imager A2 microscope (Carl Zeiss AG, Jena, Germany) and analyzed with ZEN software (version 2.1.57.1000 Lite Blue edition). Quantification of fluorescence was performed using ImageJ software (National Institutes of Health, Bethesda, MD, USA). At least three random fields per condition, including a minimum of 5 cells per field, were analyzed, and fluorescence intensity was measured per cell and expressed as normalized fluorescence units (NFUs). Experiments were performed in triplicate across three independent experiments.

### 2.12 NETosis fluorometry and immunofluorescence

Neutrophils (200,000 cells/well) were seeded in black 96-well plates and stimulated with CSNs or CCD-25Lu fibroblast supernatants for 24 h at 37 °C in a humidified atmosphere with 5% CO_2_. LPS and PMA (25 μg /mL (BioLegend^®^, Cat. No. 423302)) were used as positive controls, while untreated neutrophils served as a negative control. A total DNA control was included by lysing neutrophils with Triton X-100 (Cat. 161-0407, Bio-Rad, Hercules, CA, USA) (0.5%).

Following stimulation, plates were centrifuged at 2000 rpm for 10 min, and supernatants were carefully collected to avoid disturbing cell debris. Extracellular DNA, as a surrogate marker of NET formation, was quantified using SYTOX Green nucleic acid stain (Invitrogen™, Cat. No. s7020), which selectively binds extracellular DNA. Fluorescence was measured using a BioTek Synergy HTX plate reader at 485 nm excitation and 535 nm emission in endpoint mode. Fluorescence values were normalized against total DNA obtained by complete neutrophil lysis to control variations in cell number. Results were expressed as relative fluorescence units (RFU). Experiments were performed in triplicate across three independent experiments.

### 2.13 Statistical analysis

Each biological replicate corresponds to neutrophils isolated from an independent healthy donor. Technical triplicates were performed for each experiment unless otherwise indicated.

Statistical analyses were conducted using one-way ANOVA, followed by Dunnett's multiple comparisons test in GraphPad Prism software (version 8.0) for Windows®. Data are expressed as mean ± SD, and statistical significance was set at p < 0.05. Each experiment was conducted in at least three independent biological replicates, with technical replicates included when applicable. Significant differences are denoted as follows: *p < 0.05; **p < 0.01; ***p < 0.001; ****p < 0.0001.

## 3 Results

### 3.1 CAFs from late-stage CC are associated with higher NLR values

Primary cultures of CAFs were established from CC biopsies obtained from patients at different clinical stages. Samples were classified according to the FIGO staging system into early-stage (I–II) and late-stage (III–IV) groups.

A total of six CAF cultures were successfully obtained, all corresponding to squamous cell carcinoma. Among these, three cultures were derived from early-stage lesions (IB2–IB3), whereas the remaining three corresponded to late-stage lesions (IIIB–IIIC2).

Clinical and hematological parameters from CC patients were subsequently analyzed. Consistent with disease progression, patients with late-stage disease exhibited higher neutrophil-to-lymphocyte ratio (NLR) values than those with early-stage disease. Clinical characteristics and hematological parameters of the included patients are summarized in **Table 1**. Finally, preliminary exploratory Spearman correlation analyses were performed to evaluate the relationship between patient NLR values and the capacity of the corresponding CSN to modulate neutrophil functional responses *in vitro* (**Supplementary Figure S1**).

**Table 1:** Clinical and hematological characteristics of patients with CC included in the study.

Characteristics (n = 6)	Stage I–II (n=3)	Stage III–IV (n=3)
Age < 50 years	2 (67%)	3 (100%)
Age ≥ 50 years	1 (33%)	0 (0%)
Histology	3 SCC (100%)	3 SCC (100%)
Median Neutrophils (%)	59	75
Median Lymphocytes (%)	30.5	18.7
NLR > 3	0 (0%)	2 (66%)
NLR Values	IB2: 1.21	IIIB: 3.03
	IB2: 1.43	IIIC1: 2.90
	IB3: 2.33	IIIC2: 3.76

Patients were classified according to FIGO stage into early-stage (I–II) and late-stage (III–IV) groups. Variables include age distribution, histological subtype, hematological parameters, and neutrophil-to-lymphocyte ratio (NLR). Individual NLR values are shown for each patient according to their respective FIGO stages.

### 3.2 Characterization and transcriptomic profiling of CAFs primary cultures

Primary CAF cultures exhibited a characteristic fibroblast-like morphology, with elongated, spindle-shaped cells. These morphological features were consistently observed in CAFs derived from both early- and late-stage lesions, with no evident morphological differences between groups. To confirm the identity of CAFs, canonical markers were evaluated by immunofluorescence. CAF cultures showed positive staining for vimentin, consistent with a mesenchymal phenotype. Fibroblast activation protein (FAP) expression indicated an activated fibroblast state. Likewise, α-smooth muscle actin (α-SMA) expression is associated with cytoskeletal remodeling characteristics of activated fibroblasts. All cultures were positive for these three canonical markers, and displayed the characteristic fibroblast-like morphology, supporting the successful isolation and characterization of primary CAF cultures derived from early- and late-stage CC biopsies (**Figure 1A**).

**Figure 1:**
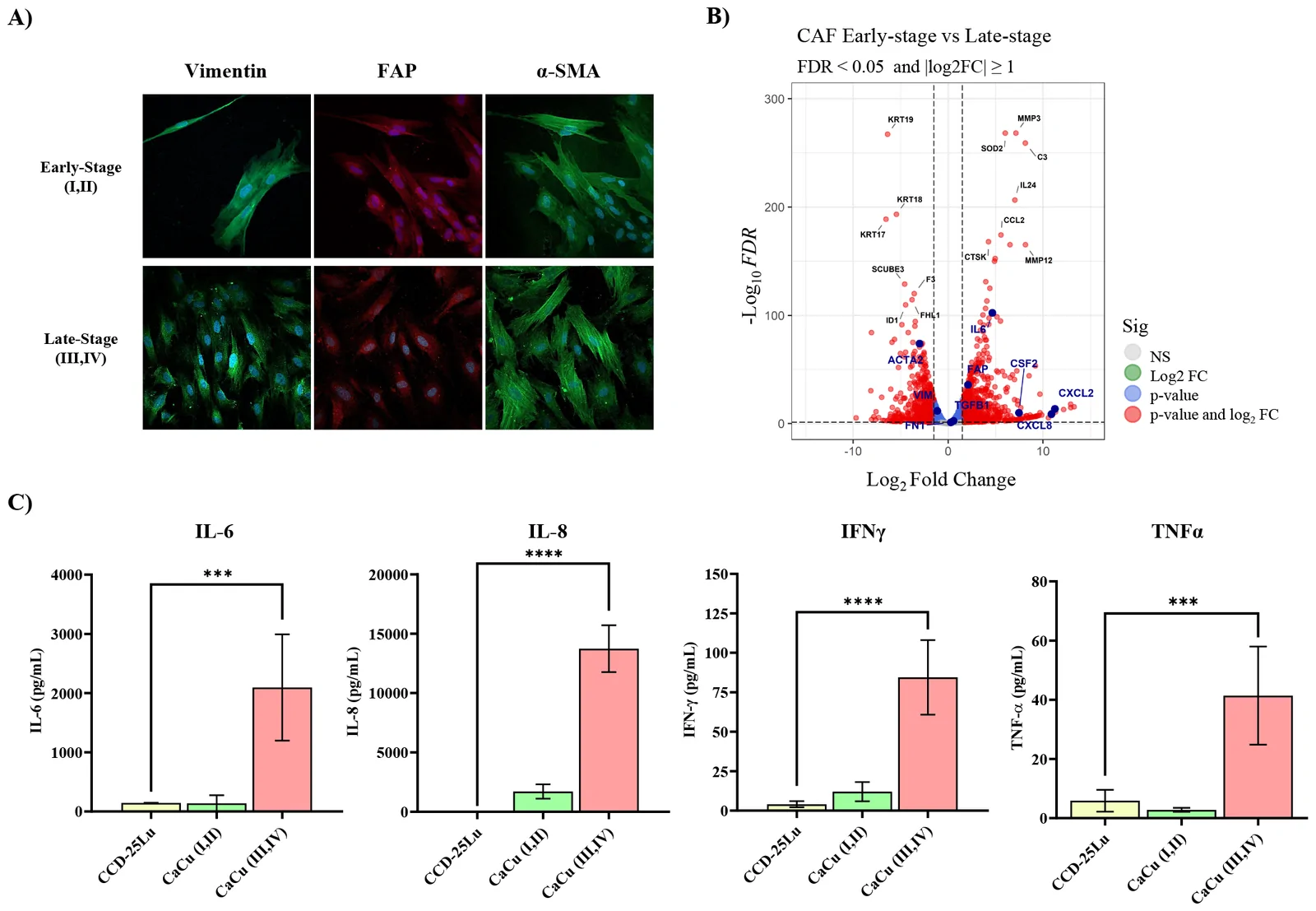
Morphological, phenotypic, transcriptomic, and secretory characterization of CAFs. **(A)** Immunofluorescence analysis of CAF-associated markers showing positive expression of vimentin (green), FAP (red), and α-SMA (green) in CAFs derived from early- and late-stage cervical cancer. Nuclei were counterstained with DAPI (blue). Images are shown at 40× magnification. **(B)** Volcano plot showing differentially expressed genes between early- and late-stage CAFs identified by RNA-seq analysis. Differentially expressed genes were defined using an FDR < 0.05 and |log2FC| ≥ 1. **(C)** Multiplex immunoassay of cytokines secreted by CSN showing increased secretion of IL-6, IL-8, IFN-γ, and TNF-α by late-stage CAFs. CCD-25Lu supernatant was used as a control for non-tumorigenic fibroblasts. Data are presented as mean ± SD. Statistical analysis was performed using one-way ANOVA followed by Dunnett's *post hoc* test. p values are indicated as ***p < 0.001 and ****p < 0.0001. CCD-25Lu: non-tumorigenic human lung fibroblast cell line; Early-stage CAFs (I/II); Late-stage CAFs (III/IV).

To further evaluate molecular differences between CAFs from distinct clinical stages, transcriptomic analysis was performed by RNA-seq. Volcano plot analysis revealed differentially expressed genes between CAFs from early- and late-stage CC. Interestingly, in this exploratory observation, late-stage CAFs tended to exhibit a transcriptomic profile characterized by increased expression of inflammatory mediators (*IL6, CXCL2, CSF2*) together with elevated proteases (*MMP3 and MMP12*) and *FAP*. In contrast, early-stage CAFs tended to exhibit higher ACTA2 expression, whereas no significant differences were observed for *VIM* expression (**Figure 1B**). Additionally, functional enrichment analyses, including Gene Ontology (GO), Kyoto Encyclopedia of Genes and Genomes (KEGG) pathway analysis (**Supplementary Figure S2**), together with Gene Set Enrichment Analysis (GSEA) of positively and negatively enriched Hallmark gene sets (**Supplementary Figure S3**), further indicated enrichment of biological processes and signaling pathways related to cytoskeletal organization, protein phosphorylation, focal adhesion, inflammatory signaling, and immune regulation. To complement these transcriptomic findings, cytokine secretion was quantified in CSN from primary CAF cultures. Consistent with these preliminary RNA-seq results, late-stage CAFs exhibited increased secretion of IL-6, IL-8, IFN-γ, and TNF-α compared with early-stage CAFs and the non-tumorigenic fibroblast CCD-25Lu (**Figure 1C**).

### 3.3 CAF supernatants promote pro-inflammatory cytokine production in neutrophils

To evaluate the effect of CSN on neutrophil activation, cytokine production was assessed in neutrophils stimulated by supernatants from early- and late-stage CAFs. Neutrophils stimulated with CAF supernatants significantly increased the production of IL-6, IL-8, IFN-γ, and TNF-α compared with untreated neutrophils (**Figure 2**). Both early- and late-stage CAF supernatants induced elevated IL-6 and IL-8 levels, comparable to those observed after LPS stimulation. Interestingly, both early- and late-stage CAFs induced increased IFN-γ levels; similar results were observed in TNF-α, with a higher increase in early-stage CAF supernatants compared to untreated neutrophils. These exploratory results support the notion that CSN promotes pro-inflammatory cytokines in neutrophils.

**Figure 2:**
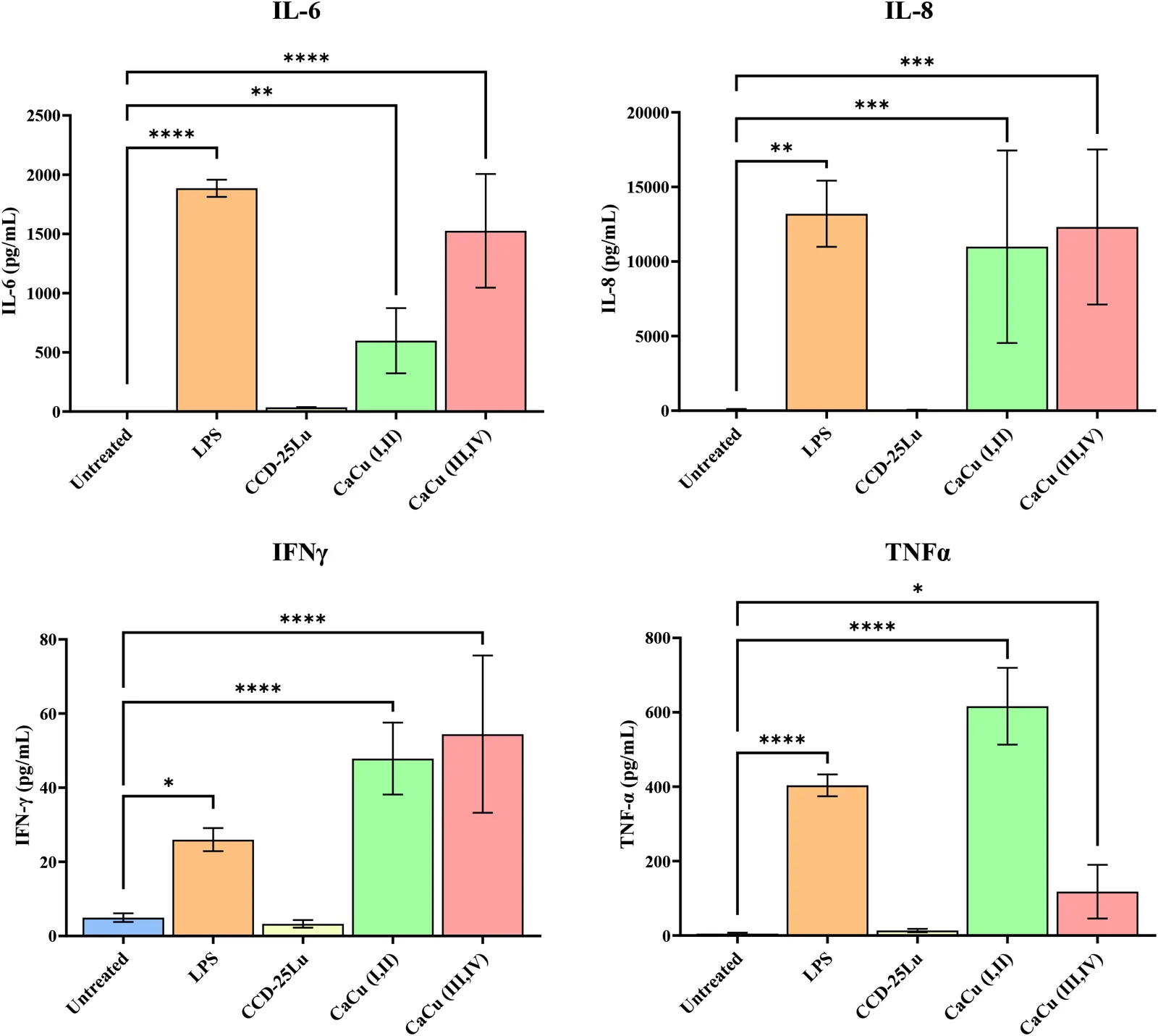
CAF supernatants induce pro-inflammatory cytokine production in neutrophils. Production of IL-6, IL-8, IFN-γ, and TNF-α was evaluated by multiplex immunoassay in neutrophils stimulated with supernatants from early- and late-stage CAFs. LPS was used as a positive control. Statistical analysis was performed using one-way ANOVA followed by Dunnett’s *post hoc* test. Data are presented as mean ± SD. *p < 0.05, **p < 0.01, ***p < 0.001, ****p < 0.0001. Untreated, unstimulated neutrophils; LPS, neutrophils stimulated with lipopolysaccharide; CCD-25Lu, neutrophils stimulated with supernatants from the non-tumorigenic fibroblast cell line; Early-stage CSN, neutrophils stimulated with supernatants from early-stage CAFs; Late-stage CSN, neutrophils stimulated with supernatants from late-stage CAFs. This stage-associated comparison should be interpreted as an exploratory observation.

### 3.4 CAF supernatants increase metabolic activity and ROS production in neutrophils

To assess the effects of CSN on neutrophil function, metabolic activity and ROS production were evaluated using MTT and ROS assays, respectively. Neutrophils stimulated with CAF supernatants exhibited significantly higher metabolic activity compared with untreated neutrophils and cells stimulated with the non-tumorigenic fibroblast reference CCD-25Lu. Among the analyzed CAF cultures, neutrophils stimulated with late-stage CAF supernatants tended to show higher metabolic activity (**Figure 3A**). Similarly, CAF stimulation significantly increased ROS production compared with untreated neutrophils (**Figure 3B**).

**Figure 3:**
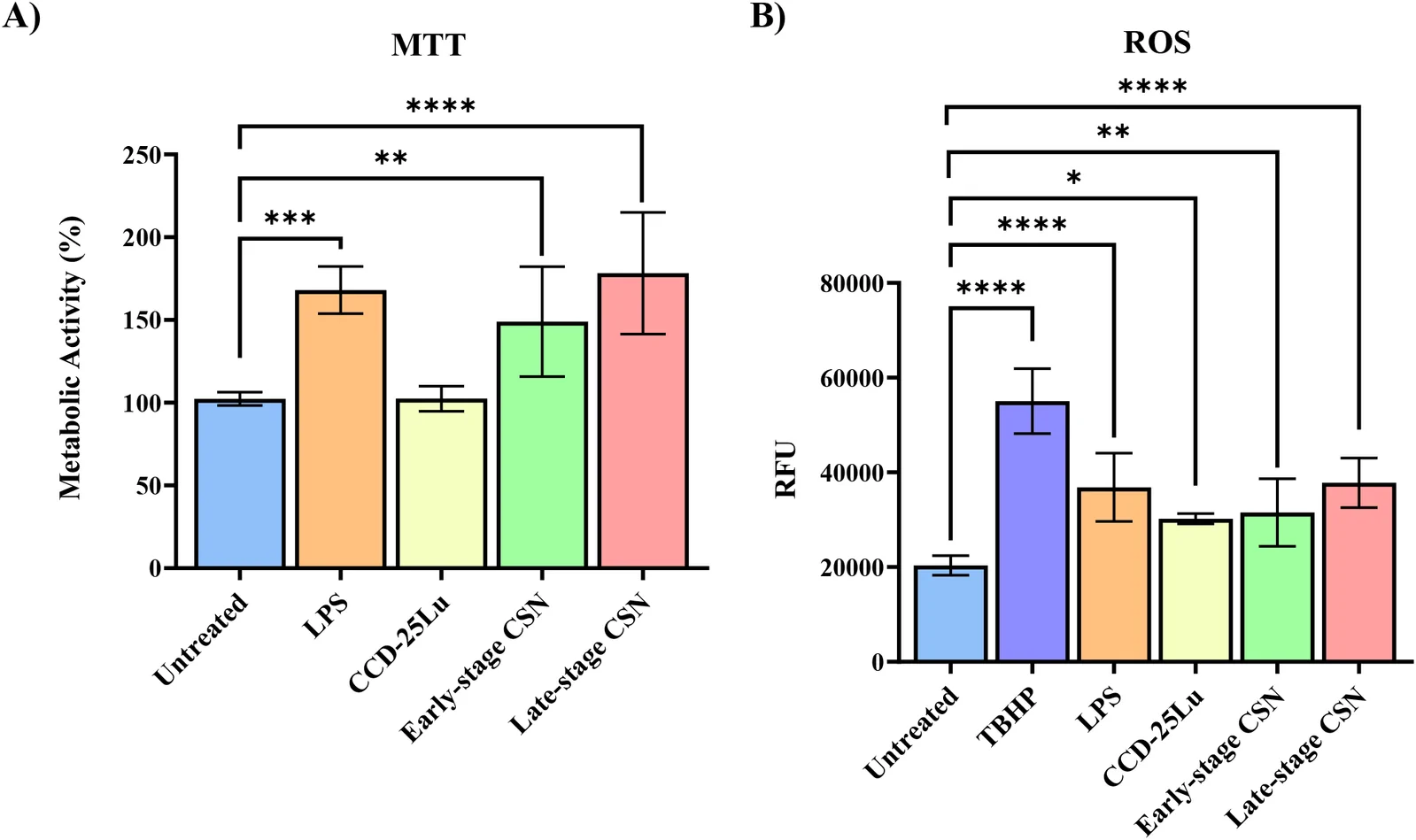
CAF supernatants increase metabolic activity and ROS production in neutrophils. Neutrophils were stimulated with supernatants from early- and late-stage CAFs to evaluate metabolic activity and ROS production. **(A)** Metabolic activity was assessed by MTT assay. **(B)** ROS production was evaluated using H2DCFDA. TBHP was used as a positive assay control. MTT results are expressed as a percentage of metabolic activity relative to untreated cells. ROS levels are expressed as relative fluorescence units (RFU). Statistical analysis was performed using one-way ANOVA followed by Dunnett’s *post hoc* test. Data are presented as mean ± SD of three independent experiments. Statistical significance is indicated as *p < 0.05, **p < 0.01, ***p < 0.001, and ****p < 0.0001. Untreated, unstimulated neutrophils; LPS, neutrophils stimulated with lipopolysaccharide; CCD-25Lu, neutrophils stimulated with supernatants from the non-tumorigenic fibroblast cell line; Early-stage CSN, neutrophils stimulated with supernatants from early-stage CAFs; Late-stage CSN, neutrophils stimulated with supernatants from late-stage CAFs; TBHP, tert-butyl hydrogen peroxide; This stage-associated comparison should be interpreted as an exploratory observation.

### 3.5 CAF supernatants reduce apoptosis in neutrophils

Neutrophil survival was evaluated by Annexin V/PI staining following stimulation with CSN from early- and late-stage CAFs. Representative flow cytometry gating strategies are shown in (**Supplementary Figure S4**). Untreated neutrophils and cells stimulated with CCD-25Lu fibroblasts displayed higher proportions of apoptotic cells, characterized by increased Annexin V-positive populations. In contrast, neutrophils stimulated with CAF supernatants showed an increased proportion of viable cells and a reduced apoptotic population (**Figure 4A**). This effect appeared more evident in neutrophils stimulated with late-stage CAF supernatants, which displayed the highest percentage of viable cells. Quantitative analysis confirmed increased neutrophil viability and reduced apoptosis in response to CAF stimulation, without substantial changes in necrotic populations (**Figure 4B**). These exploratory results indicate that CSN promotes neutrophil survival, particularly when neutrophils are stimulated with late-stage CAF supernatants.

**Figure 4:**
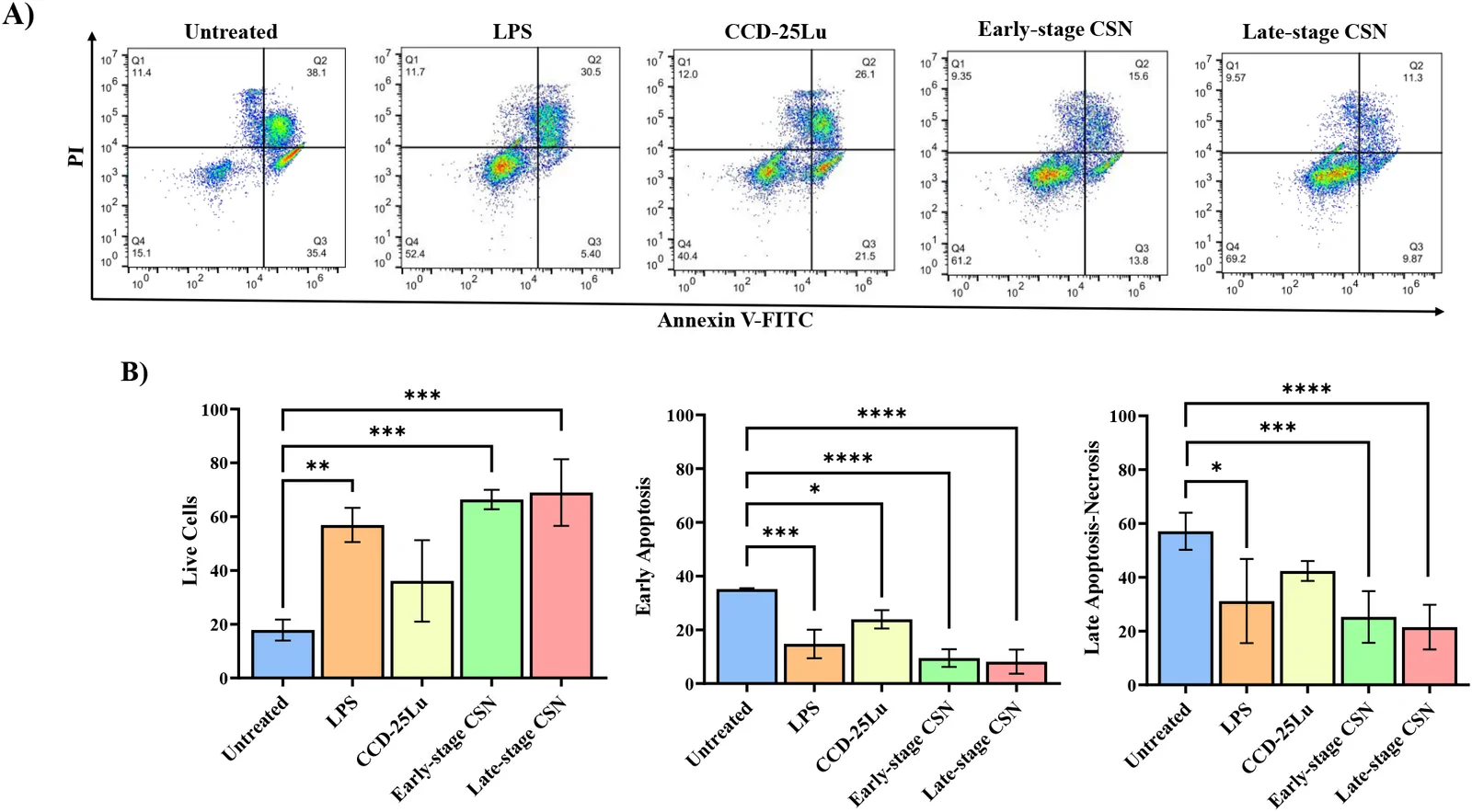
CAF supernatants enhance neutrophil survival. Apoptosis was assessed by Annexin V staining and cell death by propidium iodide (PI) staining in neutrophils stimulated with supernatants from early- and late-stage CAFs. **(A)** Representative flow cytometry dot plots showing viable, early apoptotic, late apoptotic, and necrotic neutrophil populations. **(B)** Quantification of viable, apoptotic, and necrotic cell populations under different stimulation conditions. Statistical analysis was performed using one-way ANOVA followed by Dunnett’s *post hoc* test. Data are presented as mean ± SD of three independent experiments. Statistical significance is indicated as *p < 0.05, **p < 0.01, ***p < 0.001, and ****p < 0.0001. Untreated, unstimulated neutrophils; LPS, neutrophils stimulated with lipopolysaccharide; CCD-25Lu, neutrophils stimulated with supernatants from the non-tumorigenic fibroblast cell line; Early-stage CSN, neutrophils stimulated with supernatants from early-stage CAFs; Late-stage CSN, neutrophils stimulated with supernatants from late-stage CAFs. This stage-associated comparison should be interpreted as an exploratory observation.

### 3.6 CAF supernatants increase the expression of functional markers in neutrophils.

To further characterize neutrophil activation, the expression of CD66b, MMP9, and NE were evaluated by immunofluorescence after stimulation with CAF supernatants. Neutrophils stimulated with CSN showed increased fluorescence intensity of CD66b, MMP9, and NE compared with untreated neutrophils (**Figures 5A–F**). Increased fluorescence intensity was observed in both early- and late-stage CAF stimulation conditions, with a trend toward highest expression in neutrophils stimulated with late-stage CAF supernatants. These exploratory findings indicate that CSN may promote neutrophil activation and degranulation-associated responses.

**Figure 5:**
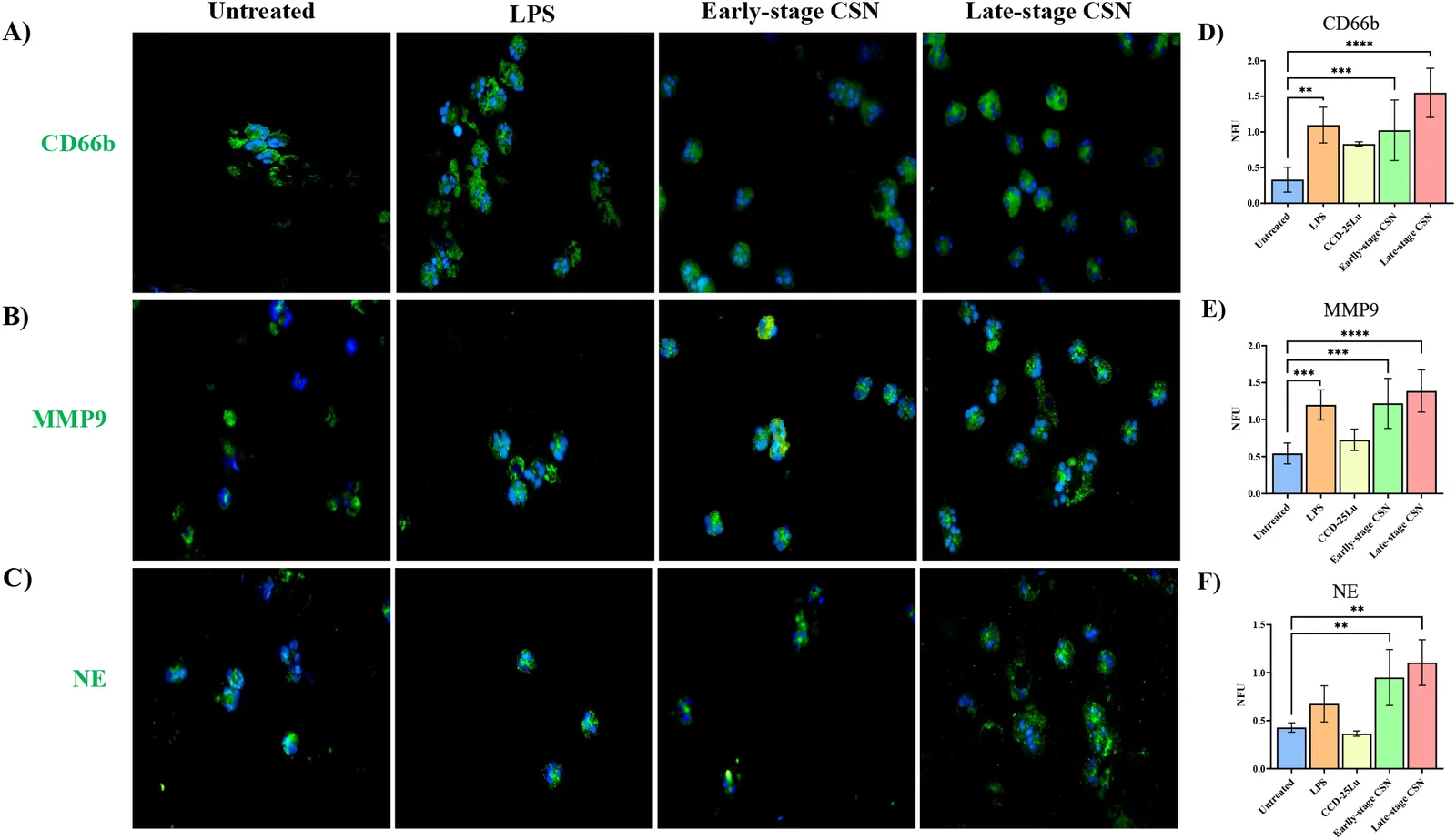
CAF supernatants increase the expression of functional markers in neutrophils. Expression of CD66b, MMP9, and neutrophil elastase (NE) was evaluated by immunofluorescence in neutrophils stimulated with supernatants from early- and late-stage CAFs. **(A–C)** Representative immunofluorescence images showing CD66b, MMP9, and NE expression (green) in neutrophils under different stimulation conditions. Nuclei were counterstained with DAPI (blue). Images are shown at 20× magnification. **(D–F)** Quantification of fluorescence intensity expressed as normalized fluorescence units (NFU) for CD66b, MMP9, and NE. Statistical analysis was performed using one-way ANOVA followed by Dunnett’s *post hoc* test. Data are presented as mean ± SD of three independent experiments. **p < 0.01, ***p < 0.001, ****p < 0.0001. Untreated, unstimulated neutrophils; LPS, neutrophils stimulated with lipopolysaccharide; CCD-25Lu, neutrophils stimulated with supernatants from the non-tumorigenic fibroblast cell line; Early-stage CSN, neutrophils stimulated with supernatants from early-stage CAFs; Late-stage CSN, neutrophils stimulated with supernatants from late-stage CAFs; TBHP, tert-butyl hydrogen peroxide; This stage-associated comparison should be interpreted as an exploratory observation.

### 3.7 CAF supernatants promote NET formation in neutrophils

Extracellular DNA release was quantified using SYTOX Green fluorescence to assess NET formation following CSN stimulation. Fluorescence microscopy analysis revealed the presence of extracellular DNA web-like structures positive for citrullinated histone H3 (H3-Cit) in stimulated neutrophils (**Figure 6A**). Neutrophils stimulated with early-stage CAF supernatants displayed more evident and extensive NET-like structures compared with untreated cells and neutrophils stimulated with late-stage CAF supernatants, which exhibited fewer and less defined extracellular structures. Consistent with these observations, quantitative analysis showed that early-stage CAF supernatants significantly increased extracellular DNA release, reaching levels comparable to those induced by PMA stimulation (**Figure 6B**). In contrast, neutrophils stimulated with late-stage CAF supernatants exhibited lower NET formation, with levels similar to those observed in CCD-25Lu-stimulated neutrophils. These exploratory observations suggest that CSN from the analyzed CAF cultures differentially modulates NET formation according to tumor stage.

**Figure 6:**
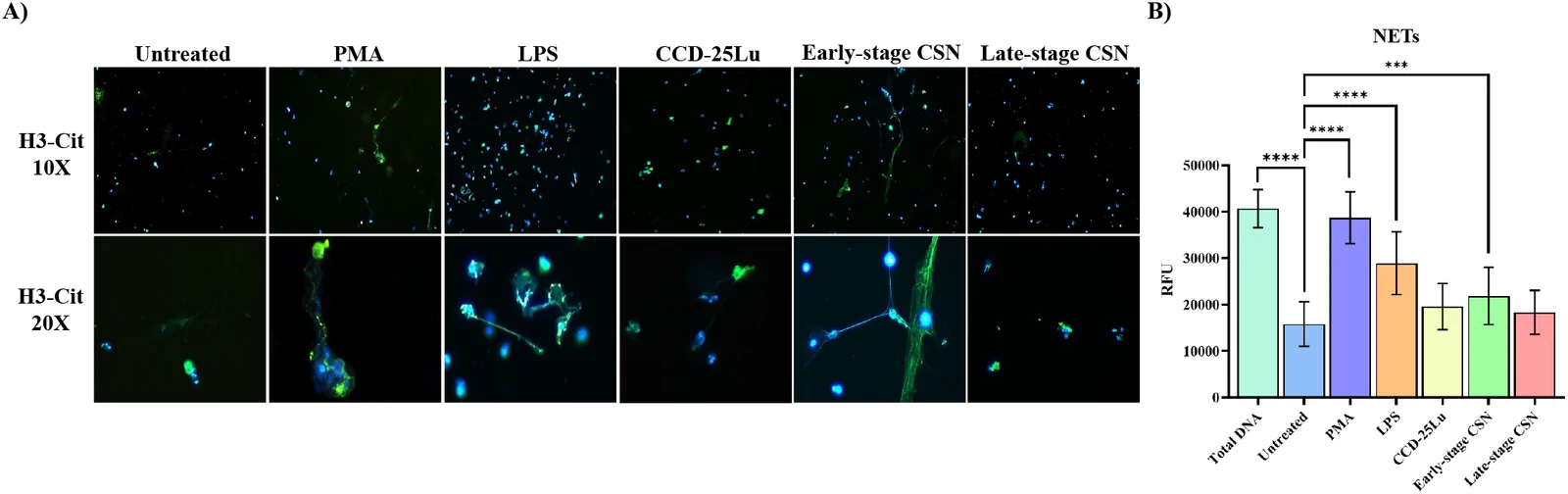
CAF supernatants promote NET formation in neutrophils. NET formation was evaluated in neutrophils stimulated with supernatants from early- and late-stage CAFs. **(A)** Representative immunofluorescence images showing extracellular DNA structures positive for citrullinated histone H3 (H3-Cit, green), and nuclei were counterstained with DAPI (blue). Images are shown at 10× and 20× magnification. **(B)** Quantification of extracellular DNA release measured by SYTOX Green fluorescence and expressed as relative fluorescence units (RFU). Total DNA was determined by complete neutrophil lysis and used as a reference for maximal DNA content. Statistical analysis was performed using one-way ANOVA followed by Dunnett’s *post hoc* test. Data are presented as mean ± SD of three independent experiments. ***p < 0.001, ****p < 0.0001. Untreated, unstimulated neutrophils; LPS, neutrophils stimulated with lipopolysaccharide; CCD-25Lu, neutrophils stimulated with supernatants from the non-tumorigenic fibroblast cell line; Early-stage CSN, neutrophils stimulated with supernatants from early-stage CAFs; Late-stage CSN, neutrophils stimulated with supernatants from late-stage CAFs; TBHP, tert-butyl hydrogen peroxide; This stage-associated comparison should be interpreted as an exploratory observation.

## 4 Discussion

In the present study, we show that soluble mediators derived from cervical CAFs functionally reprogram peripheral blood neutrophils toward an activated, pro-inflammatory functional state, characterized by enhanced survival, increased metabolic activity, pro-inflammatory cytokine secretion, increased MMP9 and NE fluorescence intensity, and reduced NET formation in response to late-stage CSN. Collectively, these functional changes may favor protumoral activities within the cervical cancer microenvironment. Our findings identify the CAF–neutrophil axis as an important and still underexplored component of the cervical cancer tumor immune microenvironment.

CAFs are among the most important cellular elements of the TME, capable of modulating the cellular and non-cellular components of the tumor through crosstalk via secreted soluble mediators. Moreover, CAFs can functionally reprogram neutrophils toward an activated, pro-inflammatory state through the release of cytokines and growth factors (28, 29). The transcriptomic analysis provided preliminary evidence of stage-differences in the remodeling of CAFs during cervical cancer progression. Late-stage CAFs showed increased expression of inflammatory mediators, including *IL6, CXCL2*, and *CSF2*, together with higher levels of the proteases *MMP3* and *MMP12* and increased *FAP* expression. By contrast, early-stage CAFs exhibited higher *ACTA2* expression than the late-stage group, whereas VIM did not differ significantly between groups. This transcriptional pattern is consistent with the recognized functional heterogeneity of CAFs, and suggests a shift from a more contractile, myofibroblast phenotype (myCAF) in early lesions toward a more inflammatory and matrix-remodeling phenotype (iCAF) in advanced disease, supporting the notion that CAFs progressively acquire protumoral functions that actively drive tumor progression rather than merely reflecting stromal expansion (30). Moreover, stage-dependent phenotypic transitions have been described in cervical CAFs, where early lesions display a more myofibroblast-like αSMA^+^/FAP^+^ profile, while advanced-stage CAFs acquire αSMA^low^/FAP^high^ phenotype, which is associated with activated CAFs, tumor progression, and stage-dependent CAF phenotypes in cervical cancer (31). The transcriptomic profile of early- and late-stage CC CAFs showed an overexpression of *FAP* in late-stage CAFs. *FAP* expression has been consistently associated with activated CAFs, tumor progression, and stage-dependent CAF phenotypes in cervical cancer. Additionally, we performed functional enrichment analyses (GO, KEGG, and GSEA), which revealed enrichment of pathways associated with inflammatory signaling, cytoskeletal remodeling, and extracellular matrix regulation (**Supplementary Figure S3**). To further corroborate the transcriptomic observations, cytokines identified by RNA-seq were additionally evaluated at the protein level in CSN from the analyzed CAF cultures. Cytokine secretion analyses provide complementary protein-level evidence and showed increased levels of IL-6 and IL-8 in late-stages suggesting that these cytokines are potentially involved in CAF-neutrophil communication. Nevertheless, these complementary data remain correlative, as the direct causal relationship with the functional responses observed in this study was not evaluated.

In cervical cancer, CAFs have been reported to promote inflammatory and matrix-remodeling programs, secreting higher amounts of IL-6; thereby inducing EMT, migration, invasion, angiogenesis, and inflammatory crosstalk within the TME (32, 33). In addition, IL-6 has been associated with neutrophil chemotaxis, survival, and STAT3 activation in other tumor models (11). Likewise, the increased IL-8 observed in late-stage CAFs may further contribute to neutrophil recruitment and priming within the cervical TME, thereby supporting the establishment of a pro-inflammatory niche, has been reported in cervical neoplasia, IL-8 has already been described as a neutrophil-priming factor, and CXCL8/IL-8 has also been linked to the regulation of neutrophil extracellular trap-related effector functions in malignant settings (34). Together, these observations identify IL-6 and IL-8 as candidate correlative mediators within the CAF secretome that may contribute to the functional reprogramming of neutrophils observed in our study. However, future studies employing cytokine neutralization or pathway inhibition will be necessary to determine the mechanistic contribution of these mediators and establish a direct causal relationship.

In addition to the local effects of CAF-derived cytokines, other studies have been associated with systemic inflammatory biomarkers. Previous studies found that higher NLR is associated with worse survival and correlates with increasing cervical lesion severity, reinforced its value as a marker of disease progression and suggesting that higher NLR values may predict poorer clinical outcomes (7, 35). Our results showed elevated NLR values in patients with advanced disease, which is consistent with the literature on cervical cancer. These preliminary exploratory findings provide indirect clinical evidence that the systemic neutrophilia observed in advanced disease may partly reflect stromal-driven reprogramming within the TME. However, given the limited patient cohort, these observations should be considered hypothesis-generating.

CAF supernatants induce a proinflammatory cytokine profile in neutrophils, with significantly higher production of IL-6, IL-8, IFN-γ, and TNF-α, which is congruent with the reprogramming described in other tumor models. The activation in neutrophils has been identified as a central mediator of their protumoral functions, including immunosuppression and promotion of tumor progression (36, 37). Consistent with these observations, other work has demonstrated that hepatocellular carcinoma CAFs activate neutrophils through the IL-6/STAT3 pathway by increasing the expression of CD66b, PD-L1, IL-8, TNF-α, and CCL2 (11). On the other hand, CAFs in pancreatic cancer promote neutrophil migration and prolong survival via IL-8 signaling (12). Our findings suggest that similar pathways may operate in cervical cancer, since García-Bernal et al, showed a high secretion of IL-6 and IL-8 by CC CAFs (38).

The neutrophil's proinflammatory profile induced by CAF was accompanied by the reduction of apoptosis, constituting a finding of considerable biological relevance. Circulating neutrophils have an inherently short half-life, and their spontaneous apoptosis is a critical regulatory mechanism to prevent tissue damage (16). The prolongation of their survival mediated by TME factors has been proposed as a prerequisite for the acquisition of protumoral functions, as it allows sustained accumulation of activated neutrophils at the tumor site (39, 40). Our results, together with the elevated metabolic activity and increased ROS production, suggest that CAFs promote the profiling of metabolically sustained neutrophils with prolonged survival and persistent effector capacity. Increased metabolic activity may reflect the energetic demands associated with cytokine secretion, degranulation, and sustained inflammatory signaling, supporting the maintenance of a protumoral neutrophil phenotype. This prolonged survival was further accompanied by increased expression of CD66b, neutrophil elastase, and MMP-9, particularly in response to late-stage CAF supernatants, indicating functional activation with enhanced degranulation capacity and increased expression of proteases. These markers are characteristic of protumoral-phenotype neutrophils capable of remodeling the extracellular matrix and facilitating tumor invasion (41, 42).

These pronounced effects induced by late-stage CAFs in our study suggest the progressive generation of highly activated neutrophils with increased expression of proteases MMP-9 and neutrophil elastase that may contribute to extracellular matrix remodeling potential as cervical cancer advances.

Interestingly, one of the most striking findings of the present study is that late-stage CAF supernatants induced less NET formation despite elevated metabolic activity and ROS production. This dissociation between ROS generation and NET release suggests that NETosis is not determined by oxidative stress alone but is actively shaped by CAF signals and the stage-specific composition of the TME. In this regard, NETs have been linked to metastasis, thrombosis, and immune evasion (13, 14, 43). Nevertheless, recent evidence suggests that the relationship between NETosis and tumor progression is more complex than initially assumed. In murine models, CAFs have been shown to induce NET formation through the secretion of amyloid-β peptide, in a ROS-dependent mechanism, and inhibition of NETosis redirects neutrophils toward a proinflammatory antitumoral phenotype (23). Accordingly, the reduced NETosis observed in our model may reflect an adaptive strategy of the advanced TME, in which neutrophils are maintained in a sustained activated state, but are preferentially redirected toward alternative effector programs, such as cytokine secretion, degranulation, and sustained ROS production, rather than terminal NET release (44, 45). This interpretation is consistent with the idea that neutrophil function changes across cervical carcinogenesis, since early lesions already show increased spontaneous NET production, whereas more advanced disease is associated with distinct neutrophil subsets linked to progression, Wnt signaling, and extracellular matrix remodeling (46, 47). Collectively, our data support a model in which cervical cancer CAFs progressively reprogram neutrophils during tumor progression, generating cells with enhanced survival, metabolic activity, increased detection of proteases, and proinflammatory signaling, but with attenuated NET production, thereby contributing to an immunosuppressive and permissive microenvironment for tumor progression (9, 48).

## 5 Limitations and strengths

This study has limitations that should be considered when interpreting the findings. First, the limited number of primary CAF cultures included (n = 3 per clinical stage) restricts the statistical power and precludes definitive conclusions regarding stage-dependent CAF biology. Although all experiments were performed using independent CAF isolates and biological replicates, the results should be interpreted as exploratory observations requiring validation in larger patient cohorts. Second, no tissue-matched normal cervical fibroblasts were available for comparison. Therefore, the CCD-25Lu human lung fibroblast cell line was included only as a non-tumorigenic fibroblast reference rather than as a normal cervical fibroblast control. Third, the experimental model was based on the *in vitro* stimulation of healthy donor neutrophils with CAF-conditioned supernatants. This approach was intentionally selected to evaluate the direct effects of CAF-derived soluble mediators under controlled experimental conditions while minimizing the influence of tumor-induced systemic alterations that may already be present in patient-derived neutrophils. Nevertheless, neutrophils from patients with cervical cancer may exhibit pre-activation, altered functional responses, or epigenetic reprogramming resulting from chronic exposure to the tumor microenvironment; therefore, the responses observed in this study may not fully recapitulate their *in vivo* phenotype. Fourth, although selected inflammatory mediators identified by RNA-seq were evaluated at the protein level using multiplex ELISA, individual transcriptomic alterations were not validated by single-gene RT-qPCR. Therefore, the transcriptomic findings should be interpreted as exploratory and will require targeted molecular validation in future studies. Finally, although the transcriptomic, secretory, and functional analyses consistently suggest that CAF-derived inflammatory mediators contribute to neutrophil functional reprogramming, the present study does not establish direct causal mechanisms. Future mechanistic studies employing cytokine neutralization, pathway inhibition, or complementary functional rescue approaches, together with validation in patient-derived neutrophils, paired normal cervical fibroblasts, and *in vivo* models, will be required to determine the direct contribution of individual CAF-derived mediators, including IL-6 and IL-8, to neutrophil functional reprogramming and to validate the present findings.

Despite these limitations, this study has several strengths. To our knowledge, it is the first to comprehensively characterize the effects of primary cervical cancer-derived CAF-conditioned supernatants from different clinical stages on multiple neutrophil functional responses, including metabolism, survival, ROS production, cytokine secretion, protease-associated expression, and NET formation. Moreover, the integration of transcriptomic, functional enrichment (GO, KEGG, and GSEA), secretory, and functional analyses provides a comprehensive overview of the CAF–neutrophil interaction in cervical cancer. Although exploratory, these findings identify the CAF–neutrophil axis as a promising area for future investigation and provide a rationale for mechanistic studies aimed at developing stromal-targeted immunomodulatory strategies in cervical cancer.

## Data Availability

The RNA-seq data generated for this study have been deposited in the NCBI Gene Expression Omnibus (GEO) under accession number GSE347724.
