# Supplementary material for: Cancer-associated fibroblasts from cervical cancer modulate protumoral neutrophil functions: evidence from a pilot exploratory study
Source: Front Immunol. 2026 Oct 9;17:1900567. doi: 10.3389/fimmu.2026.1900567 (PMC13690724; doi:10.3389/fimmu.2026.1900567)
Supplement: Supplementary file 1 [file SupplementaryFile1.docx]

Supplementary Material

# 1 Supplementary Figures

Supplementary Figure S1. Supplementary Figure S1. Exploratory Spearman correlation matrix between patients' neutrophil-to-lymphocyte ratio (NLR) and CAF-induced neutrophil functional responses. Correlation analyses were performed using the NLR values of the patients from whom CAFs were isolated and the corresponding functional responses induced by their CAF-conditioned supernatants in healthy donor neutrophils, including reactive oxygen species (ROS), neutrophil survival, NET formation, IL-6, and IL-8 production. Numbers within each cell indicate Spearman's correlation coefficients (r). Blue and red represent positive and negative correlations, respectively, whereas color intensity reflects the strength of the association. * P < 0.05; ** P < 0.01 denote statistically significant correlations. These exploratory correlations provide indirect clinical evidence and should be interpreted as preliminary and hypothesis-generating.

Supplementary Figure S2. Supplementary Figure S2. Functional enrichment analyses of differentially expressed genes in cervical cancer-associated fibroblasts (CAFs). (A) KEGG pathway enrichment analysis showing the most significantly enriched pathways associated with cytoskeletal remodeling, protein phosphorylation, focal adhesion, cell cycle regulation, NF-κB signaling, JAK-STAT signaling, and viral carcinogenesis. (B) Gene Ontology (GO) enrichment analysis showing the most significantly enriched biological processes, molecular functions, and cellular components among differentially expressed genes. Bubble size indicates the number of genes associated with each term or pathway, whereas color intensity represents enrichment significance (−log10 adjusted P value).

Supplementary Figure S3. Supplementary Figure S3. Gene Set Enrichment Analysis (GSEA) of Hallmark gene sets in cervical cancer-associated fibroblasts (CAFs). (A) Positively enriched Hallmark gene sets (positive normalized enrichment score, NES). (B) Negatively enriched Hallmark gene sets (negative normalized enrichment score, NES). Bubble size represents the number of genes contributing to each enriched gene set, whereas color intensity corresponds to the false discovery rate (FDR). Gene sets are ranked according to their normalized enrichment score (NES).

Supplementary Figure 4. Flow Cytometry Gating Strategy. Cells were identified and selected based on their size and internal complexity characteristics using FSC-A (Forward Scatter Area) and SSC-A (Side Scatter Area) parameters, excluding debris and non-cellular events (left panel). Subsequently, doublet discrimination was performed using the relationship between FSC-A and FSC-H, selecting only single cells to avoid the inclusion of cell aggregates in the analysis (middle panel). Finally, single cells were analyzed by Annexin V-FITC and PI staining to determine cell viability and cell death (right panel).
